# Nitrogen Reserve Pools in Two *Miscanthus* × *giganteus* Genotypes under Contrasting N Managements

**DOI:** 10.3389/fpls.2017.01618

**Published:** 2017-09-20

**Authors:** Ryan M. Dierking, Damian J. Allen, Suzanne M. Cunningham, Sylvie M. Brouder, Jeffrey J. Volenec

**Affiliations:** ^1^Actagro, LLC, Fresno CA, United States; ^2^Mendel BioEnergy Seeds, Mendel Biotechnology Inc., Hayward CA, United States; ^3^Department of Agronomy, Purdue University, West Lafayette IN, United States

**Keywords:** *Miscanthus*, nitrogen, reserves, genotype, rhizome, growth

## Abstract

Nitrogen (N) reserves in vegetative tissues contribute N to regrowth of *Miscanthus* × *giganteus* shoots in spring, but our understanding of how N fertilization and plant genotype affect this process is incomplete. Our specific objectives were to: (1) determine how N fertilizer management impacts accumulation of dry matter and N among aboveground and belowground tissues and organs; (2) understand how changes in N management and tissue N concentration influence seasonal fluctuations in concentrations of buffer-soluble proteins and amino acids in putative storage organs including rhizomes and roots; and (3) characterize genotypic variability and genotype × N interactions for N reserve accumulation and use among *Miscanthus* × *giganteus* genotypes. Established plots of the IL Clone and Nagara-sib population were fertilized with 0–0, 0–150, 75–75, 150–0, and 150–150 kg N ha^-1^ where the first numeral denotes the N rate applied in 2011 (Year 1) and the second number denotes the N rate applied in 2012 (Year 2). Rhizomes, roots, stembases, and shoots were sampled at 6-week intervals between March and August and then in November at dormancy. Concentrations of N, soluble protein and amino-N increased in all tissues with fertilizer N application. With the exception of rhizome amino-N, concentrations of these N pools in roots and rhizomes declined as plants resumed growth in spring and increased sharply between August and November as growth slowed. Losses in shoot and stembase N mass between August and November were similar to total N accumulation in roots and rhizomes during this interval. Compared to the unfertilized control, specific N managements enhanced growth of above- and belowground tissues. The IL Clone generally had greater biomass yield of all organs than the Nagara-sib; the exception being shoot biomass in November when extensive leaf senescence reduce yield of the IL Clone. High biomass yields were obtained with 75 kg N ha^-1^ applied annually rather than semi-annual N applications of 150 kg N^-1^ ha that depended on N recycling from roots/rhizomes as a supplemental N source.

## Introduction

Second generation lignocellulosic biofuels are expected to include plant species that produce large amounts of high-fiber biomass with a reduction in fertilizer input, especially nitrogen (N) (Heaton E. et al., 2004; Wang et al., 2012). This reduction in N input is critical to system net energy balance as the synthesis of inorganic N fertilizer consumes vast amounts of natural gas (Erisman et al., 2008). Lower N fertilizer input for perennial bioenergy crops may be plausible, in part, due to their ability to accumulate N reserves in storage organs that supplement soil N pools in providing N to shoots when growth is initiated in spring and resumes after biomass harvest in summer (Volenec et al., 1996). However, our understanding of N reserves in the context of N use efficiency (NUE) of *Miscanthus* × *giganteus* is fragmented and incomplete.

The NUE of *Miscanthus* has been examined from several perspectives. Beale and Long (1997) reported high biomass production per unit N based upon the low N removal in harvested aboveground biomass. They reported late-season translocation of N from biomass to rhizomes as one factor contributing to this high NUE. These results were later confirmed by Heaton et al. (2009) who showed large late-season declines in aboveground biomass N concentrations. Using ^15^N Christian et al. (2006) verified intra-plant N cycling from shoots to other tissues with rhizomes being a large late-season N sink. Significant amounts of rhizome N were transferred to shoots in subsequent growing seasons suggesting that this N was serving as a reserve pool. However, large amounts of ^15^N remained in rhizomes through Year 3 indicating that some rhizome N pools may not be readily mobilized.

Previous work with perennial plants used for forage and pasture can inform hypotheses regarding N storage in *Miscanthus* and other perennial biomass crops. For example, several forage legumes accumulate vast quantities of N in taproots during autumn that are subsequently used for shoot growth initiation in spring and shoot regrowth after defoliation in summer (Hendershot and Volenec, 1993a,b; Barber et al., 1996; Li et al., 1996); times when N from N_2_ fixation is inadequate to meet plant N needs (Vance et al., 1979). In alfalfa (*Medicago sativa* L.) this N accumulates primarily as vegetative storage proteins (VSPs, Cunningham and Volenec, 1996) that, like seed storage proteins, are rapidly degraded and translocated to regrowing shoots to meet their N needs. Like alfalfa, white clover (*Trifolium repens* L.) also appears to accumulate species-specific VSPs during autumn that are mobilized when growth resumes in spring (Corre et al., 1996). However, not all perennial legumes used for forage accumulate VSPs as a storage N form. While taproots of red clover (*T. pratanse* L.), sweetclover [*Melilotus officinalis* (L.) Lam.] and birdsfoot trefoil (*Lotus corniculatus* L.) accumulate N in autumn that is depleted when shoot growth resumes in spring, these species do not appear to accumulate taproot VSPs (Li et al., 1996). Reserve N accumulation is not limited to perennial legumes. Uptake of nitrate and ammonium from the soil by forage grasses is severely reduced by defoliation in summer (Bakken et al., 1998; Louahlia et al., 1999). These plants mobilize leaf sheath and root N pools to regrowing leaf blades (Ourry et al., 1989, 1990). Furthermore, when soil N supply is adequate, VSPs accumulate in sheath tissues of ryegrass (*Lolium perenne* L.) and these are subsequently mobilized to new leaves during post-defoliation regrowth in summer (Louahlia et al., 1999). Like perennial legumes, however, not all perennial grasses accumulate VSPs as the primary N reserve in vegetative tissues. For example, *Calamagrostis epigejos* utilizes both free amino acids and soluble proteins in roots and stubble soluble proteins as the principle N sources during regrowth after defoliation (Kavanova and Gloser, 2005).

Potential contribution of both reserve N and mineralized soil N pools to shoot N of regrowing *Miscanthus* increases uncertainty regarding N fertilizer requirements when compared to conventional annual row crops like maize. Depending on soil characteristics, prevailing environment, and stand age, yield responses of *Miscanthus* to N fertilizer in research plots vary widely from unresponsive to N fertilizer (Lewandowski et al., 2000; Christian et al., 2008; Arundale et al., 2014; Larsen et al., 2014; Finnan and Burke, 2016) to requiring >100 kg N ha^-1^ annually (Lewandowski et al., 2000; Khanna et al., 2008; Pedroso et al., 2014; Dierking et al., 2016). A recent Extension guide for growing *Miscanthus* biomass in the central United States suggests applying 80 to 130 kg N ha^-1^ to replace the N removed in a 30 t ha^-1^ biomass yield (Heaton et al., 2016). Improving our understanding the nature and extent of N cycling in *Miscanthus* should inform future N recommendations and improve both NUE and ultimately system net energy balance.

Finally, virtually all of the published research has focused on N responses of the “IL Clone” of *Miscanthus* × *giganteus*. However, there are substantial differences among other *Miscanthus* × *giganteus* ecotypes and populations for most phenotypic traits including cell wall structure and biomass combustion efficiencies, flowering, leaf senescence, mineral concentrations/contents, and yield differences (Jørgensen, 1997; Clifton-Brown and Lewandowski, 2002; Hodgson et al., 2010; Allison et al., 2011; da Costa et al., 2014; Iqbal and Lewandowski, 2014). The progenitor species to *Miscanthus* × *giganteus* are known outcrossing species and possess high levels of heterozygosity and vary phenotypically (Zhao et al., 2013). For example, Aurangzeb (2012) evaluated nine distinct *Miscanthus* × *giganteus* genotypes derived from novel crosses among progenitor lines and observed significant morphological differences in crown size and leaf structure. Jeżowski (2008) working with novel *Miscanthus* genotypes during establishment also observed differences in crown and tiller morphology. Less is known regarding genotypic differences in mineral nutrition including N storage and its impact on NUE. Here we report how N management strategies alter N storage patterns and pools, and subsequent N mobilization to regrowing shoots of two *Miscanthus* × *giganteus* lines previously shown to differ in biomass, NUE, and late-season leaf retention (Dierking et al., 2016). Our specific objectives were to: (1) determine how N fertilizer management impacts accumulation of dry matter (DM) and N among aboveground and belowground tissues and organs; (2) understand how changes in N management and tissue N concentration influence seasonal fluctuations in concentrations of buffer-soluble proteins and amino acids in putative storage organs including rhizomes and roots; and 3) characterize genotypic variability and genotype × N interactions for N reserve accumulation and use among *Miscanthus* × *giganteus* genotypes.

## Materials and Methods

### Location, Fertilization, and Genetic Materials

For a full description of the site, N management strategies, and plant material see Dierking et al. (2016). The scope of that original experiment was reduced in magnitude as described below in order to facilitate the intensive sampling associated with this study. Briefly, the experiment was planted at Lafayette, IN (40.484096, -86.815827) on a Billett loam in 2010 at a population density of 19,760 plants ha^-1^. Two of four contrasting *Miscanthus* × *giganteus* genotypes (IL Clone; open pollinated (OP) Nagara-sib) were selected for study based on pronounced variation in aboveground morphology and previous differences in NUE (Dierking et al., 2016). A subset of the most extreme five of seven N management treatments was selected for this study including 0–0, 0–150, 75–75, 150–0, and 150–150 kg N ha^-1^ where the first numeral denotes the N rate applied in 2011 (Year 1) and the second number denotes the N rate applied in 2012 (Year 2). Nitrogen was hand-applied as Agrotain^TM^-coated urea on June 1 and May 2 of Years 1 and 2, respectively. Each genotype-N management combination was replicated three times. Mean monthly temperatures and precipitation were recorded at a weather station on the experimental site (**Table 1**). The 50-year weather data record was obtained from the Purdue University Airport located 15 km southwest of the study site.

**Table 1:** Monthly average temperatures and precipitation for the duration of the study.

	Temperature, °C			Precipitation, mm		
Month	2011	2012	Average	2011	2012	Average
January	-5	0	-3.9	18.5	58.7	48.7
February	0	1.7	-1.7	67.6	31.5	41.9
March	5.6	13.9	4.6	54.6	48.5	65.3
April	12.8	12.2	11	200.4	58.2	94.5
May	17.8	19.4	16.6	30.7	76	99.6
June	22.8	22.2	21.8	164.9	28.5	107.7
July	26.7	27.2	23.8	85.6	23.1	95.8
August	23.9	22.2	22.8	124.5	125.5	92
September	18.3	17.2	18.9	95.8	99.8	73.4
October	12.8	10.6	12.3	63.3	141.2	65.8
November	8.9	4.4	5.7	142.5	20.3	74.2
December	2.8	2.8	-0.5	84.1	64.5	66.3


*Also included are the 50-year average temperatures and precipitation for this location*.

### Above and Belowground Sampling

A single plant from the outer two rows of four row plots was collected from each plot every 6 weeks starting in March and ending in August of 2012. A final sample was collect just prior to machine harvest in November 2012. Border effects were minimized by surrounding each plot with other *Miscanthus* × *giganteus* plants established at the same population and planted on the same day. During sampling, aboveground shoot biomass was removed approximately 15 cm above the soil surface. Biomass was weighed immediately, coarsely chopped and a subsample (about 500 g fresh weight) collected. This subsample was weighed, dried in a forced-air oven at 60°C until constant weight was attained and the percent moisture used to calculate plant biomass yield. After the aboveground shoot tissues were collected, all rhizomes and associated roots for the plant were excavated with shovels (dimension means ± standard errors: surface area, 2028 ± 62 cm^2^; depth: 15 ± 0.3 cm; volume: 31,262 cm^3^ ± 1383 cm^3^). These tissues were cleaned under a stream of cold tap water and separated into roots, rhizomes, and stem bases (shoot tissue between the soil surface and where shoot removal occurred). Tissues were blotted dry, weighed immediately to determine fresh weights, and a representative subsample transferred to paper bags. These samples were placed in -80°C freezer for at least 24 h before being transferred to -4°C. Tissues were held at -4°C until lyophilized (FreeZone 12 freeze dryer, Labconco Corporation, Kansas City, MO, United States). Freeze-dried tissues were weighed and percent moisture used to calculate DM yields of below-ground tissues and stembases. Tissues were initially ground to pass a 6-mm screen (Wiley mill, Thomas Scientific, Swedesboro, NJ, United States), then re-ground to pass a 1-mm screen using a cyclone sample mill (Udy Corp., Fort Collins, CO, United States) for laboratory analysis. Lyophilized tissues were stored at -4°C.

### Nitrogen, Buffer-Soluble Protein, and Amino Acid-N Analysis

Tissues were analyzed for total N concentration using a flash combustion elemental analyzer (Flash EA 1112 Series, Thermo Fisher Scientific, Netherlands). Procedures to determine buffer-soluble protein and amino acid-N concentrations in rhizome, root, and stem base tissues were conducted at temperatures between 0 and 4°C unless otherwise stated. Proteins were extracted by suspending 30 mg of tissue and equal masses of insoluble polyvinylpolypyrrolidone (Sigma Chemical Co., St. Louis; product P6755) in 1 mL of 100 mM NaPO_4_ buffer (pH 6.8) containing 10 mM 2-mercaptoethanol and 1 mM phenylmethylsulfonyl fluoride. Tubes were kept on ice while being vortexed four times at 5-min intervals. Samples were centrifuged at 14,000 × *g* for 10 min. Soluble protein in the supernatant was estimated using the protein dye-binding method of Bradford (1976). Bovine serum albumin was used as a standard. Concentration of buffer-soluble amino acids in the supernatant was determined using ninhydrin with glycine as the standard (Rosen, 1957).

### Yield and N Content Calculations

Total aboveground mass was estimated by adding stembase dry mass to the dry mass of the shoots, while the total belowground biomass was estimated as the sum of the dry masses of the roots and rhizomes. The N content was calculated as the product of N concentration and dry mass of each tissue. These values were summed to determine the total belowground and aboveground N mass per plant. Total DM and N content per hectare in November at harvest was determined as the product of the soil surface area occupied per plant and plant population.

### Statistical Analysis

The experimental design was a randomized complete-block design with a factorial arrangement of five N managements and two genotypes replicated three times. Plots were sampled repeatedly up to six times (tissue dependent). A split-plot-in-time analysis of variance analysis was used to partition variation into genotype, N management, replicate, and month effects and interactions using Minitab (17.3.1). Genotype and N management main effects were tested using the genotype × replicate and N management × replicate interaction terms, respectively. Harvest effects and interactions were tested with the mean square error term (Cochran and Cox, 1957). Where the *F*-test was significant (*P* < 0.05) the least significant difference (LSD) was calculated unless otherwise indicated (**Table 2**).

**Table 2:** Summary of analysis of variance results showing the effects of nitrogen management (N), genotype (G), month of harvest (Harv.) and corresponding interactions on dry wt., and concentrations of N, protein, and amino-N in shoot, stem base, root and rhizome tissues of *Miscanthus* × *giganteus*.

		Main effect or interaction									
Tissue	Trait	N	G	Harv.	N × G	N × Harv.	G × Harv.	N × G × Harv.
Shoot	Dry weight	ns	ns	^∗∗^	ns	ns	^∗∗^	ns
	N	^∗∗^	ns	^∗∗^	ns	^∗^	ns	ns
Stem base	Dry weight	^∗^	ns	^∗∗^	ns	†	^∗∗^	ns
	N	^∗∗^	ns	^∗∗^	ns	†	ns	ns
	Protein	^∗^	ns	^∗∗^	ns	ns	^∗∗^	ns
	Amino-N	^∗∗^	ns	^∗∗^	ns	^∗∗^	ns	ns
Root	Dry weight	ns	^∗^	^∗∗^	ns	ns	ns	ns
	N	^∗∗^	^∗^	^∗∗^	^∗∗^	^∗^	ns	ns
	Protein	^∗∗^	ns	^∗∗^	†	^∗∗^	ns	†
	Amino-N	^∗∗^	^∗^	^∗∗^	^∗∗^	^∗∗^	ns	ns
Rhizome	Dry weight	ns	^∗∗^	^∗∗^	ns	ns	ns	ns
	N	^∗∗^	^∗^	^∗∗^	^∗^	^∗∗^	ns	ns
	Protein	^∗∗^	ns	^∗∗^	^∗∗^	^∗∗^	ns	†
	Amino-N	^∗∗^	ns	^∗∗^	^∗^	^∗∗^	ns	ns


*Significance levels (^†^, ^∗^, ^∗∗^) denoted significant differences at *P* < 0.10, 0.05, and 0.01 levels of probability, respectively*.

## Results

### Weather

Temperatures during the experiment were similar to the long-term average with the exception of warmer than normal temperatures in March in 2012 and July of both years (**Table 1**). Precipitation in 2011 was greater than normal in April, June, and November, while January and May of 2011 were dry. In 2012 June, July, and November were drier than normal and October wetter than the 50-year average.

### Effects of Genotype and N Management on Tissue Dry Matter Yields

**FIGURE 1:**
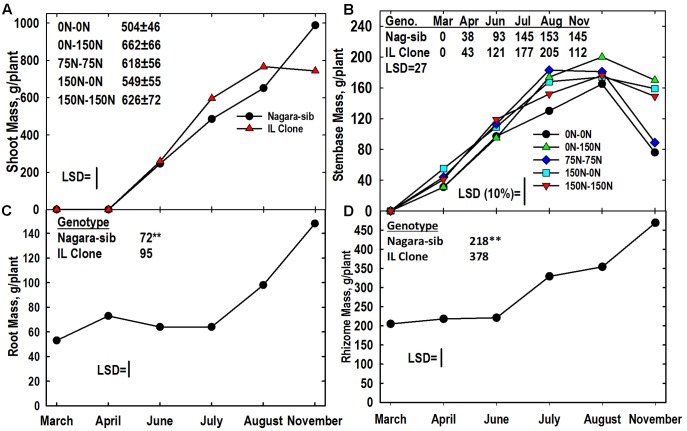
Tissue dry matter yields of two *Miscanthus* genotypes (Nagara-sib, IL Clone) as influenced by nitrogen (N) management. **(A)** Means and least significant difference (LSD) for the genotype × harvest interaction on shoot biomass yield and the main effect means ± standard error of N management on shoot mass (tabulated). **(B)** Stembase means and LSDs for the N management × harvest interaction (plotted) and genotype × harvest interaction (tabulated) effects. The N treatments (all in kg N ha^-1^) included: no N application either year (0N–0N), N application only in Year 1 (150N–0N) or Year 2 (0N–150N), or N application both years of the study (75N–75N; 150N–150N). **(C)** Means and LSDs for the main effects of harvest (plotted) and genotype (tabulated) on root mass. **(D)** Means and LSDs for the main effects of harvest (plotted) and genotype (tabulated) on rhizome mass. Tissue sampling commenced in March of Year 2. The LSDs are provided at *P* < 0.05. In **(C,D)** genotypic differences designated with ^∗∗^*P* < 0.01.

A significant genotype × harvest interaction was observed. Shoot mass per plant increased in both genotypes, but mass was greater in the IL Clone in July and August, whereas the Nagara-sib had greater shoot mass in November (**Figure 1A**). The N treatment main effect for shoot yield was tested with less precision in the split-plot analysis and was not statistically significant (*P* > 0.05). Trends in shoot mass averaged across genotypes and harvests varied with N management ranging from 504 and 549 g/plant, respectively, for the 0–0 and 150–0 treatments not receiving N in Year 2 to over 600 g/plant for treatments receiving N in Year 2 (**Figure 1A**).

The N treatment x harvest and genotype x harvest interactions were significant for stembase mass per plant (**Table 2**). Stembases of the IL Clone were larger from June through August when compared to the Nagara-sib, but the IL Clone had lower stembase mass in November (**Figure 1B**). Stembases were not present at the March harvest, but increased rapidly during April and July. Stembase mass of the 0–0 N treatment was lower than that of the 0–150 N treatment from July to November, and the 150–0 and 75–75 treatments in July. By November stembase mass of the 0–0 and 75–75 N treatments declined markedly and were lower than the other N treatments. Averaged over genotypes and harvests stembase mass of the 0–0 N treatment was lower than that of all other N treatments (data not shown).

Nitrogen management did not impact root and rhizome mass significantly (**Table 2**). Significant genotype and harvest main effects were observed for both the root and rhizome mass per plant. Averaged across N treatments and harvests, the IL Clone had greater root and rhizome mass than the Nagara-sib (**Figures 1C,D**). Root mass initially increased from March to April followed by large increases between July and November. Rhizome mass also exhibited a large increase from June to November.

### Tissue Nitrogen

**FIGURE 2:**
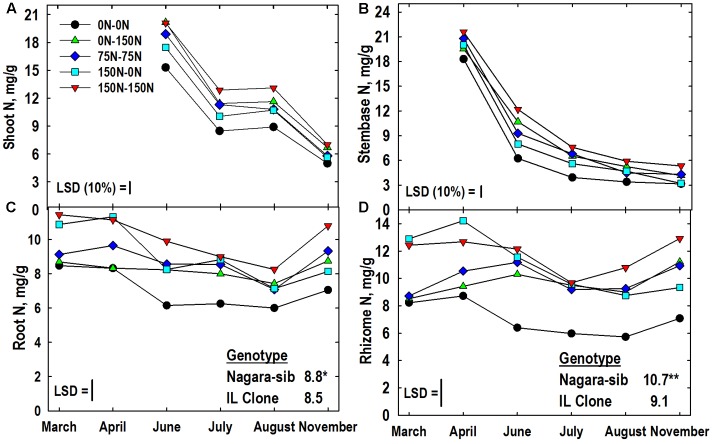
Tissue N concentrations of two *Miscanthus* genotypes (Nagara-sib, IL Clone) as influenced by N management. **(A)** Shoot N means and least significant differences (LSDs) for the N management × harvest interaction effect. **(B)** N management × harvest interaction means for stembase N concentrations. **(C)** Root N means and LSDs for the N management × harvest interaction effect (plotted) and genotype main effect (tabulated). **(D)** Rhizome N means and LSDs for the N management × harvest interaction effect (plotted) and genotype main effect (tabulated). The N treatments (all in kg N ha^-1^) included: no N application either year (0N–0N), N application only in Year 1 (150N–0N) or Year 2 (0N–150N), or N application both years of the study (75N–75N; 150N–150N). Tissue sampling commenced in March of Year 2. The LSDs are provided at *P* < 0.05 unless otherwise noted. Genotypic differences designated with ^∗^*P* < 0.05, ^∗∗^*P* < 0.01, respectively.

The main effect of genotype was significant for root, and rhizome N concentrations (**Figures 2C,D**). The magnitude of these differences was relatively small, but consistent, with the Nagara-sib having higher N concentrations than the IL Clone. All tissues had a significant N management × harvest interaction, however, the significance level was at the 10% level of probability for shoot and stembase tissues. As expected shoot N concentrations in June were greatest in the 150–150 and 0–150 N treatments that received the highest N fertilization rates in Year 2 and lowest in plants from the 0–0 N control plots. Shoot N concentrations declined by nearly 50% in July but the relative rankings of the N treatments remained similar through June. Shoot N concentrations in August were similar to July values, but by November shoot N declined and only plots receiving 150 kg N ha^-1^ in Year 2 had higher concentrations than the 0–0 control plots.

Stembase N concentrations declined rapidly between April and July as new shoots initially emerged, but eventually extended past this lower portion of the canopy (**Figure 2B**). Stembase N concentrations of the 0–0 plots in April were lower than all treatments that had received N the previous year (**Figure 2B**). While N concentrations in stembases of the 0–0 plots remained lower than other treatments throughout the growing season, stembase N concentrations of the 0–150 N treatment increased relative to other treatments and were similar to the 150–150 N treatment in June. By November stembase concentrations of the 150–0 N treatment declined to values similar to the 0–0 control, and both of these were lower than the 150–150 N treatment

As anticipated root N concentrations in March and April were highest in plots fertilized with high N in Year 1 (150–0, 150–150) and lowest in plots not receiving N in Year 1 (0–0, 0–150) (**Figure 2C**). Application of N in Year 2 increased N concentrations in roots of the 0–150 treatment in June when compared to the 0–0 control, while root N concentration of the unfertilized 150–0 N treatment declined. The 0–0 treatment had lower root N than the other treatments in July. In August and November the 0–0 and 150–150 N treatment had the lowest and highest root N concentrations, respectively, with the other N treatments intermediate. Irrespective of N application, all treatments exhibited a general increase in root N concentration between August and November. The genotype × N treatment interaction was significant for root N concentration (**Table 3**). Root N concentration of the IL Clone was greater than the Nagara-sib in the 0–150 N treatment, whereas the reverse was true at the 150–150 N treatment.

**Table 3:** Influence of nitrogen (N) management on concentrations of N, buffer-soluble protein, and amino acid-N in roots and rhizomes of two *Miscanthus* genotypes (Nag., Nagara-sib; IL, IL Clone).

	Root						Rhizome					
	N mg/g DW		Protein mg/g DW		Amino-N μM/g DW		N mg/g DW		Protein mg/g DW		Amino-N μM/g DW	
N Mgmt	Nag.	IL	Nag.	IL	Nag.	IL	Nag.	IL	Nag.	IL	Nag.	IL
0N–0N	7.2	6.8	2.4	2.4	50	42	7.7	6.3	3.5	3.6	103	81
0N–150N	7.9	8.6	2.7	3.0	65	79	9.7	9.5	4.0	5.0	164	186
75N–75N	8.9	8.6	2.9	3.1	85	76	10.8	9.2	4.9	5.0	199	163
150N–0N	9.4	8.8	3.2	3.1	98	76	11.8	10.2	5.4	4.9	204	187
150N–150N	10.7	9.5	3.8	3.4	134	91	13.6	10.3	5.9	5.2	275	217
LSD	0.7		0.3^†^		14		1.1		0.5		32	

*^†^LSD at the 10% level of probability. Data were averaged harvests in Year 2.Nitrogen treatments (all in kg N ha^-*1*^) included: no N application either year (0N–0N), N application only in Year 1 (150N–0N) or Year 2 (0N–150N), or N application both years of the study (75N–75N; 150N–150N). The least significant difference (LSD) at *P* ≤ 0.05 level of probability is provided unless indicated otherwise*.

Like roots, rhizome N concentrations in March and April reflected Year 1 N management with higher concentrations in the 150–150 and 150–0 N treatments (**Figure 2D**). Rhizome N concentrations from June to August were lower in the 0–0 N treatment when compared to the other treatments. Between August and November rhizome N concentrations increased in treatments that receive N in Year 2. By comparison, the 0–0 and 150–0 N treatments exhibited only a slight increase in rhizome N between August and November. In general rhizome N concentrations were depleted between April and July, and N subsequently re-accumulated by November. Rhizome N concentration also exhibited a significant genotype × N treatment interaction (**Table 3**). Averaged across harvests, rhizome N concentrations of the Nagara-sib generally increased with increasing N fertilization (e.g., 0–0 to 75–75 to 150–150), while concentration of N in rhizomes of the IL Clone were higher than the 0–0 control plots when N was applied, but similar among the N treatments themselves. In general, the Nagara-sib had higher rhizome N concentrations than the IL Clone with the exception of the 0–150 N treatment.

### Buffer-Soluble Protein and Amino Acid-N Pools

**FIGURE 3:**
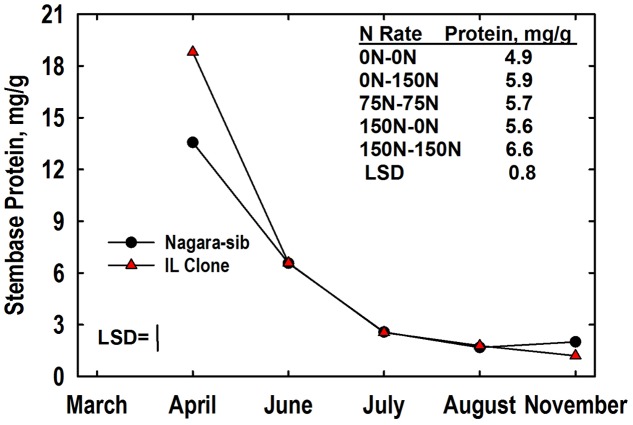
Stembase protein concentrations of *Miscanthus* genotypes as influenced by N management. Means and least significant differences (LSDs, *P* < 0.05) for the genotype (Nagara-sib, IL Clone) × harvest interaction effect (plotted) and the main effect means of N management (tabulated) are provided. The N treatments (all in kg N ha^-1^) included: no N application either year (0N–0N), N application only in Year 1 (150N–0N) or Year 2 (0N–150N), or N application both years of the study (75N–75N; 150N–150N). Stembase sampling commenced in April of Year 2.

Total buffer-soluble proteins were analyzed in this study as a surrogate for yet uncharacterized VSPs in storage organs of this species. Like N, stembase protein concentrations declined markedly between April and July harvests (**Figure 3**). There was a significant genotype × harvest interaction for stembase protein. Concentrations of protein in stembases of the IL Clone were higher than those of the Nagara-sib in April but stembase protein levels of genotypes were similar at all subsequent harvests. Averaged over genotypes and harvests, the main effect of N management on stembase protein concentration also was significant. Stembase protein concentrations of plants fertilized with N in Year 2 (75–75, 0–150, 150–150) were higher than the 0–0 control N treatment. In addition, the stembase protein concentrations of the150–150 N treatment were higher than those of the 75–75 and 150–0 N treatments. Shoot tissues were not analyzed for concentrations of buffer-soluble protein and amino acid N.

**FIGURE 4:**
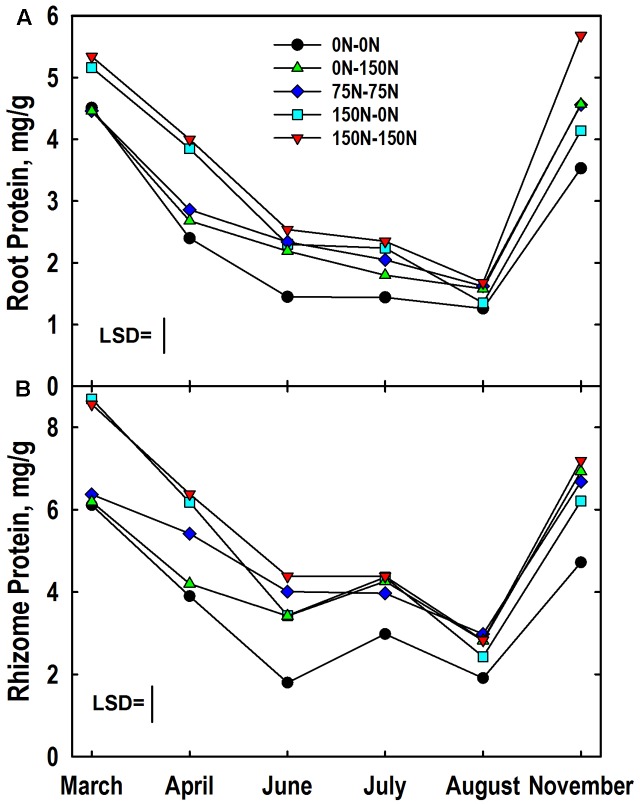
Protein concentrations in roots **(A)** and rhizomes **(B)** of *Miscanthus* as influenced by N management. Means and least significant differences (LSD, *P* < 0.05) for the N management × harvest interaction are provided. The N treatments (all in kg N ha^-1^) included: no N application either year (0N–0N), N application only in Year 1 (150N–0N) or Year 2 (0N–150N), or N application both years of the study (75N–75N; 150N–150N). Tissue sampling commenced in March of Year 2.

A significant harvest × N treatment interaction was observed for root protein concentration. Like root N, protein concentrations were highest in roots of plants fertilized with 150 kg N ha^-1^ in Year 1 (**Figure 4A**). Root protein concentrations declined in all N treatments in April, with continued rapid decline in the 0–0, 150–0, and 150–150 N treatments until June at which time the 0–0 N treatment had a lower root protein concentration than the other N treatments. Low root protein concentrations were observed in August, and there was no difference associated with N treatment. However, protein concentrations increased markedly between August and November with the highest concentrations observed in roots of plants fertilized with N in Year 2. Root protein levels in November were equal to or higher than those observed the previous March for all treatments where N was provided in Year 2. In contrast, root protein levels in November were lower than March concentrations for the 0–0 and 150–0 N treatments. Root protein concentration also exhibited a significant genotype × N treatment interaction (**Table 3**). The IL Clone had greater protein concentrations in roots of plants in the 0–150 N treatment whereas the Nagara-sib had higher root protein than the IL Clone for plants in the 150–150 N treatment.

The harvest × N treatment interaction also was significant for rhizome protein concentration. March protein concentrations were higher in rhizomes of the 150–0 and 150–150 N treatments when compared to the other N treatments (**Figure 4B**). Rhizome protein concentrations declined in all treatments until June, but the extent of decline was altered by N fertilizer application. For example, rhizome protein concentrations of the 0–0 and 0–150 N treatments were similar to each other at March and April harvests, but N application in early May slowed the decline in rhizome protein levels of the 0–150 N treatment when plots were sampled in June. Likewise, the protein concentrations for the 150–0 and 150–150 N treatments were similar in March, but by the June harvest protein concentrations in rhizomes of the 150–0 N treatment were more extensively depleted than the 150–150 N treatment that received N fertilizer in May. Rhizome protein concentrations remained low in the 0–0 N treatment from June to August. Large increases in rhizome protein concentration occurred for all N treatments between August and November, and for the 75–75 and 0–150 treatments protein concentrations returned to levels observed the previous March. The genotype × N treatment interaction also was significant for rhizome protein concentration (**Table 3**). Protein concentrations in rhizomes of the Nagara-sib increased incrementally as N treatment increased from 0–0 to 150–150. In contrast, protein concentrations in rhizomes of the IL Clone were elevated to a similar level over the 0–0 N treatment irrespective of the N fertilizer application rate or timing.

**FIGURE 5:**
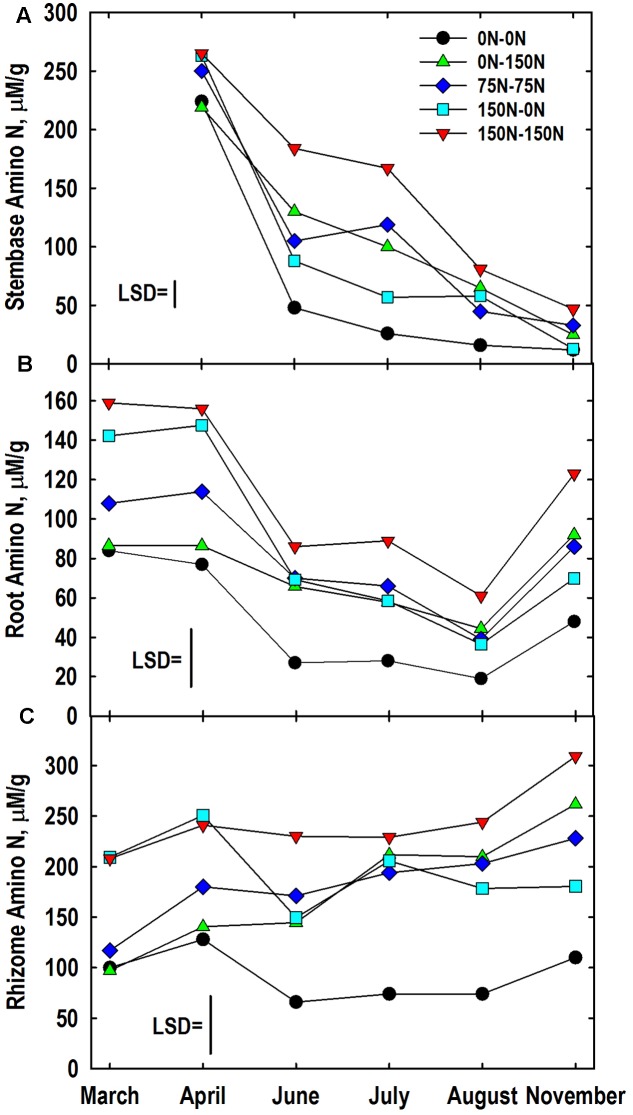
Amino-N concentrations in stembase **(A)**, root **(B)**, and rhizome **(C)** tissues of *Miscanthus* as influenced by N management. Means and least significant differences (LSD, *P* < 0.05) for the N management × harvest interaction are provided. Nitrogen treatments (all in kg N ha^-1^) included: no N application either year (0N–0N), N application only in Year 1 (150N–0N) or Year 2 (0N–150N), or N application both years of the study (75N–75N; 150N–150N). Tissue sampling commenced in March (roots, rhizomes) or April (stembases) of Year 2.

The harvest × N treatment interaction was significant for amino-N concentrations of stembases (**Figure 5A**). Stembase amino-N concentrations in April of the 0–0 and 0–150 N treatments were lower than treatments that had received N in Year 1. Stembase amino-N declined by June, especially in the 0–0 and 150–0 N treatments. Application of N in Year 2 slowed the decline in amino-N concentrations in the 75–75, and 150–150 N treatments resulting in large differences among treatments in June and July. By the November sampling concentrations of amino-N in the 0–0 and 150–0 N treatments were similar and lower than the 150–150 N treatment.

The harvest × N treatment interaction also was significant for root amino-N concentrations (**Figure 5B**). Large differences in root amino-N were observed in March and April, with the 0–0 and 0–150 N treatments being lower than plants fertilized with 150 kg N ha^-1^ the previous year. Root amino-N declined in all treatments by June and remained low through August when only the 0–0 and 150–150 N treatments differed. Root amino-N accumulated between August and November, especially in plots fertilized with N in Year 2. The genotype x N treatment interaction also was significant for root amino-N concentration (**Table 3**). Root amino-N of the Nagara-sib generally increased incrementally as N treatment increased from 0–0 to 150–150 N. Amino-N in roots of the IL Clone increased from 42 μM/g for the 0–0 N treatment to 76 to 79 μM/g for 0–150, 75–75, and 150–0 N treatments. Roots of the IL Clone in the 150–150 N treatment contained the highest amino-N concentrations (91 μM/g), but this was less than that observed for the Nagara-sib provided this N regime.

Concentration of amino-N in rhizomes of the 150–0 and 150–150 N treatments were greater in March and April when compared to the other treatments (**Figure 5C**). Between April and June large reductions in rhizome amino-N concentrations were observed for the 0–0 and 150–0 N treatments, while amino N concentrations in rhizomes of the other treatments remained unchanged. By comparison, amino-N concentrations in rhizomes of the 0–150 N treatment that was similar to the 0–0 N treatment in March and April were higher than these plots in June and at subsequent samplings. Amino-N accumulated in rhizomes between August and November in all but the 150–0 N treatment with final concentrations reflecting N fertilizer applications in Year 2. The genotype × N treatment interaction also was significant for rhizome amino-N (**Table 3**). Averaged across harvests, rhizome amino-N concentrations generally increased in both genotypes with N fertilization. Amino-N concentrations of the Nagara-sib were greater than the IL Clone when fertilized with N both years (75–75 and 150–150 N treatments).

### Nitrogen Contents in above- and belowground Tissues

**FIGURE 6:**
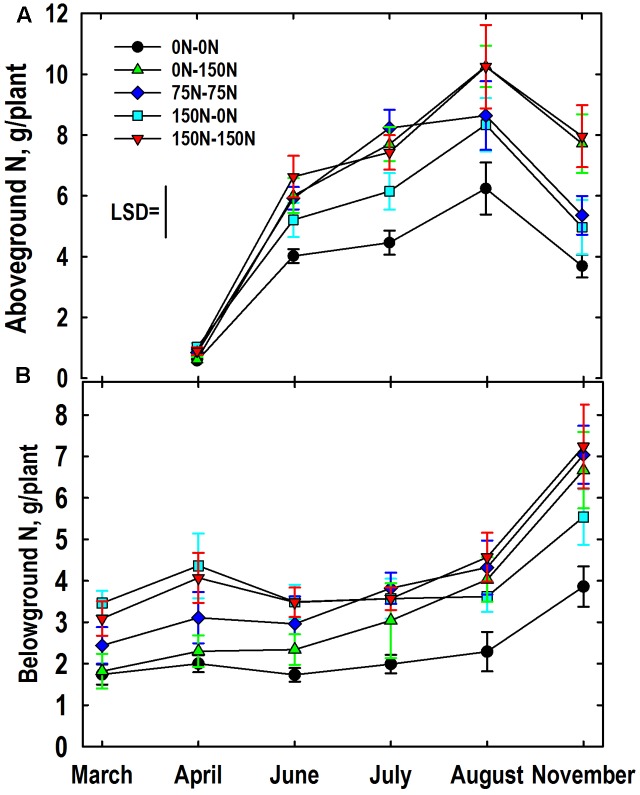
Mass of N in aboveground (**A**, shoots and stembases) and belowground (**B**, roots and rhizomes) as influenced by N management. Data were averaged over *Miscanthus* genotypes. Nitrogen treatments (all in kg N ha^-1^) included: no N application either year (0N–0N), N application only in Year 1 (150N–0N) or Year 2 (0N–150N), or N application both years of the study (75N–75N; 150N–150N). Tissue sampling commenced in March (roots, rhizomes) or April (stembases, shoots) of Year 2. The least significant difference (LSD) is provided for comparison of aboveground means (*P* < 0.05). The N treatment × harvest interaction was not significant (*P* = 0.20) for belowground N mass so standard errors are provided.

The genotype main effect on aboveground N mass (N concentration × tissue mass; summed for shoots and stembases) was not significant. The harvest × N treatment interaction for aboveground N mass was significant. Averaged over genotypes aboveground N mass was similar for all N treatments in April (**Figure 6A**). As expected aboveground N mass increased markedly from April to July at which time the 0–0 N treatment had less accumulated N than all other treatments, and the 150–0 N treatment had less than the 75–75 N treatment. Aboveground N mass increased in all plots between July and August except the 75–75 N treatment where the aboveground N mass was similar to the 150–0 N treatment. From August and November aboveground N mass declined and treatment differences established in August were largely maintained.

The main effect of genotype on belowground N mass (N concentration × tissue mass; summed for rhizomes and roots) was significant with the IL Clone averaging more N mass belowground than the Nagara-sib (4.24 vs. 2.99 g/plant, respectively). The main effects of N treatment and harvest on belowground N mass were also highly significant. Averaged over N treatments and genotypes, belowground N mass was lowest in March (2.5 g/plant); an amount similar to values observed in April (3.2 g/plant) and June (2.8 g/plant). However, belowground N masses in July (3.4 g/plant) were higher than those observed in March, and those observed in August (3.8 g/plant) exceeded belowground N masses observed in both March and June. Belowground N mass in November (6.0 g/plant) was higher than all other observed values. The harvest × N treatment interaction was not significant (*P* = 0.20) because trends over harvests were generally similar among N treatments. Nevertheless, the harvest × N treatment means with their standard errors are provided (**Figure 6B**) in order to be consistent with presentation of other N and tissue mass data (**Figures 1–5, 6A**). Means differing by twice the standard error or more are considered significantly different. Belowground N mass in March and April of the 0–0 and 0–150 N treatments were lower than the 150–0 and 150–150 N treatments, with the 75–75 N treatment intermediate. Belowground N mass of the 0–150 N treatment was greater than the 0–0 N treatment in June and subsequent harvest reflecting the large N fertilizer application this treatment received in Year 2. Likewise, belowground N mass of the 150–0 N treatment that was initially high in March did not increase between June and August ultimately placing this N treatment intermediate between the 0–0 N control and the other N treatments all of which received N in Year 2. All plants accumulated N belowground between August and November irrespective of N treatment, however, the trajectory of N accumulation was greater in plots fertilized with N in Year 2.

## Discussion

### Yield and Tissue N

This experiment varied N management over two growing seasons to alter N concentrations and N pools in putative storage organs in order to inform the relationships between uptake, accumulation, and remobilization of N, and plant growth/biomass yield. To broaden the inference space we used the commonly grown IL Clone of *Miscanthus* and the lesser studied Nagara-sib germplasm that have previously been shown to differ in yield, N use efficiency, and late-season leaf senescence (Dierking et al., 2016). Application of N fertilizer increased tissue N concentrations of all organs, and when compared to the unfertilized 0–0 N control plants, and there was a trend to enhance biomass yield (**Figures 1, 2**). Several reports also have indicated greater biomass yield of *Miscanthus* in response to N fertilization (Himken et al., 1997; Heaton E.A. et al., 2004; Christian et al., 2008; Miguez et al., 2008; Maughan et al., 2012). However, others have found no response of *Miscanthus* to N fertilizer (Lewandowski et al., 2000; Christian et al., 2008; Arundale et al., 2014; Larsen et al., 2014; Finnan and Burke, 2016).

Although root and rhizome N concentrations of the 0–150 N treatment increased significantly after N fertilizer application at the beginning of Year 2 (**Figures 2C,D**) season-average dry weights of these tissues did not increase in response to this N (**Table 2**). Wiesler et al. (1997) did observe that shoot growth of *Miscanthus sinensis* was enhanced immediately after N application whereas root and rhizome growth were less responsive to N fertilization. Amougou et al. (2011) also observed no increase in root and rhizome mass when *Miscanthus* was fertilized with 120 kg N ha^-1^despite a large increase in tissue N concentrations. These authors reported total N mass accumulation in belowground organs that ranged from 94 to nearly 300 kg N ha^-1^ depending on management and time of sampling. Similar variation and absolute levels of belowground N mass accumulation have been reported in other studies (Neukirchen et al., 1999; Christian et al., 2006; Dohleman et al., 2012; Dufossé et al., 2014). By comparison, when N mass per plant data (**Figure 6B**) are scaled to the 19760 plant ha^-1^ plant populations, we estimated that belowground tissues contained between 34 (0–0 treatment) and 71 (150–0 treatment) kg N ha^-1^ in March. Belowground N mass increased to 77 and 142 kg N ha^-1^ by November for the 0–0 and 150–150 N treatments, respectively. Part of this November increase in belowground N is presumed to originate from N translocated from senescing aboveground tissues (Beale and Long, 1997; Heaton et al., 2009; Cadoux et al., 2012). Based on plant populations and N mass plant^-1^, N content of the aboveground tissues declined on average 56 kg N ha^-1^ (range 46 to 67 kg N ha^-1^) between August and November (**Figure 6A**). During this time interval, belowground biomass accumulated an average of 46 kg N ha^-1^ (range 31 to 57 kg N ha^-1^). Assuming no N leached from tissues and minimal additional N uptake from August to November as reported by Burks (2013), these changes represent an 82% recovery of N lost by aboveground tissues in belowground storage organs. Our predicted N transfer values are similar to those reported by Christian et al. (2006) who calculated that 60 and 74 kg N ha^-1^ moved from aboveground to belowground organs of 2- and 3-year-old plants, respectively. Strullu et al. (2011) reported slightly higher estimates of N transfer from aboveground to belowground tissues (up to 145 kg N ha^-1^). The higher values were for late-season harvests that had higher biomass yields than observed in our study.

The traditional fertilization strategy of annual application of N (75–75, 150–150 N treatments) along with the 0–0 control treatment provided useful context for understanding the effectiveness of alternate-year N fertilizer applications (150–0, 0–150). In general, tissue N concentrations were lowest for the 0–0 N treatment, highest for the 150–150 N treatment, and intermediate for the 75–75 N treatment (**Figure 2**). Plants provided high N only in Year 1 (150–0 N treatment) had high N concentrations and masses in roots and rhizomes initially that were depleted during the following growing season (**Figure 6**). In contrast, plants provided high N only in Year 2 began with low N concentrations and masses in roots and rhizomes, but these increased quickly following N application; a response previously reported by others (Amougou et al., 2011; Strullu et al., 2011). Biomass yield of plants receiving N only in Year 1 (150–0 N treatment) was similar to the 0–0 N treatment (**Figure 1A**) suggesting that previously accumulated N in belowground organs in Year 1 was not sufficient to meet the shoot N needs of plants in Year 2. This conclusion is also supported by the reduced aboveground N mass of the 150–0 N treatment in June and July when compared to the N-fertilized treatments even though belowground biomass of the 150–0 N treatments contains large amounts of N (**Figure 6**). Annual application of 75 kg N ha^-1^ in this study resulted in both high yield and well-developed roots and rhizomes compared to alternate-year N application at double the rate that rely on N recycling/remobilization in the unfertilized year. Himken et al. (1997) also recommended annual applications of 50 to 70 kg N ha^-1^ for high yields of well-established stands of *Miscanthus* in Europe; a fertilizer management strategy endorsed by others, especially when large amounts of biomass are removed from the field (Heaton et al., 2009; Strullu et al., 2011; Dohleman et al., 2012; Ferchaud et al., 2016).

### Protein and Amino N Fluxes and N Management Strategies

Although root and rhizome N concentrations were generally similar (**Figures 2C,D**), rhizomes tended to have greater concentrations of amino-N and buffer-soluble protein when compared to roots (**Figures 4, 5**). This, along with their three-fold greater mass when compared to roots (**Figures 1C,D**) indicates that fluxes in N reserve pools to/from rhizomes represent a greater N mass flow and likely contribute more N to shoot growth in spring than N reserve pools in roots. Root and rhizome protein concentrations both declined in spring when growth resumed and accumulated in autumn as growth subsided and plants acclimated for winter (**Figure 4**). This general pattern agrees with previous research on vegetative storage proteins in perennials (Hendershot and Volenec, 1993b; Avice et al., 2003). As expected, protein concentrations of both tissues in March of Year 2 generally reflected Year 1 N management whereas protein concentrations in November of Year 2 responded to that season’s N management. Addition of N fertilizer has previously been shown to increase protein accumulation in storage organs of perennials and enhanced subsequent shoot growth rates after defoliation (Volenec et al., 1996; Avice et al., 2003). Likewise, preloading N in belowground storage organs of *Miscanthus* with N in Year 1 (150–0 N treatment; **Figure 6B**) also enhanced initial aboveground growth in Year 2 (**Figure 6A**) when compared to the 0–0 N treatment control plants; however, the enhanced initial growth was not sustained and season-average shoot mass of the 150–0 N treatment (549 g/plant) was similar to the 0–0 N treatment (504 g/plant) (**Figure 1A**).

Amino-N concentrations in March and November in both roots and rhizomes were closely associated with N management imposed over the 2-year period (**Figure 5**). Reduction in amino-N concentrations of roots and rhizomes of N-deprived (0–0 and 150–0 N treatments) plants suggest this pool is an important contributor of N to shoot growth in spring. Gloser (2002, 2005) also reported extensive loss of amino acids from rhizomes of *C. epigejos* in spring followed by a gradual increase in this N pool from July through November. He concluded that amino acids in rhizomes, roots and stem bases have a central role in N storage, winter survival, and spring growth of this species. While root amino N concentrations underwent a depletion-accumulation cycle similar to root and rhizome protein (**Figure 4**), amino-N concentrations of rhizomes of plants receiving N in Year 2 gradually increased from March to November. This contrasting trend in the amino-N pool, when compared to the rhizome protein pool, may be misleading as it does not capture potential rapid turnover of and flux through the amino-N pool. This is similar to reserve carbon pools in summer in perennial plants where starch reserves vary markedly following defoliation but only modest changes in sugar concentrations are observed (Gallagher et al., 1997; Teixeira et al., 2007). Labeling studies are necessary to inform the rate and extent of turnover of the amino-N pool in roots, but especially rhizomes of *Miscanthus*. Frak et al. (2002) used ^15^N labeling to understand the dynamics and temporal succession of individual amino acids involved in N remobilization in walnut (*Juglans nigra* ×*regia*) trees. Others have used dual-labeling with both ^15^N and ^13^C to understand the magnitude of contribution of taproot C and N reserves, including amino acids, to shoot C and N nutrition and ultimately biomass growth (Avice et al., 1996).

### Genotypic Effects

Previous work (Dierking et al., 2016) revealed significant differences in growth and N use efficiency among these *Miscanthus* genotypes. Genotypes differed in all biomass traits (**Figure 1**) including a significant reduction in shoot biomass between August and November for the IL Clone as leaves senesced, while leaves of the Nagara-sib remained largely intact (Dierking et al., 2016). Mass of roots and rhizomes were generally greater for the IL Clone (**Figures 1C,D**) and this greater mass may have diluted root and rhizome N pools and contributed to the lower N concentrations of these tissues (**Figures 2C,D**).

Genotypes differed in concentrations of N, protein, and amino-N in rhizomes and these differences occasionally interacted with N management (**Tables 2, 3**). For example, when compared to the IL Clone, N pools in roots and rhizomes of Nagara-sib were greater in the 150–150 N treatment, whereas the reverse was generally observed for the 0–150 N treatment (**Table 3**). Both genotype- and fertilizer-induced differences in accumulation of protein and amino-N in storage organs has been previously reported for several perennial species (Haagenson et al., 2003; Gloser, 2005; Patton et al., 2007; Berg et al., 2009; Lissbrant et al., 2010). Initial shoot growth in spring and shoot regrowth after defoliation were generally positively associated with accumulation of protein and/or amino-N in storage organs in these studies. In this study shoot mass of the IL Clone tended to be greater than that of the Nagara-sib in July and August (**Figure 1A**) indicating that, despite lower N pool concentrations in roots and rhizomes (**Figures 2C,D** and **Table 3**), the nearly two-fold greater mass of rhizomes (**Figure 1D**) could have contributed more N mass from reserves to initial shoot growth. Additional work with a larger array of *Miscanthus* genotypes may inform the relationships among N reserve pools and genotypic differences in growth and stress tolerance.

## Conclusion

As expected, both N concentration and content of above- and belowground plant tissues were greatly influenced by N management. The N accumulated as amino-N and protein in roots and rhizomes, with the latter organ accumulating the most reserve N mass. Belowground N pools accumulated the previous year were depleted when shoot growth resumed in spring, but alone, were insufficient to maintain rapid shoot growth into summer. Highest biomass yields were associated with moderate amounts of N (e.g., 75 kg N ha^-1^) applied annually. Finally, application of N fertilizer to unfertilized *Miscanthus* (e.g., 0–150 treatment) results in rapid recovery of both tissue N concentrations levels and biomass accumulation rate.

## Author Contributions

RD, DA, SB, and JV designed and planned the experiment. RD and SC performed the experiments and laboratory analyses. RD, SB, and JV analyzed the data. RD drafted the manuscript. DA, SC, SB, and JV reviewed/revised the manuscript; all authors approved the final version to be published.

## Conflict of Interest Statement

The authors declare that the research was conducted in the absence of any commercial or financial relationships that could be construed as a potential conflict of interest.
